# Important knowledge gaps among pastoralists on causes and treatment of udder health problems in livestock in southern Ethiopia: results of qualitative investigation

**DOI:** 10.1186/s12917-017-1222-1

**Published:** 2017-10-23

**Authors:** Kebede Amenu, Barbara Szonyi, Delia Grace, Barbara Wieland

**Affiliations:** 1 School of Veterinary Medicine, Hawassa University, P.O. Box 5, Hawassa, Ethiopia; 2 International Livestock Research Institute, P. O. Box 5689, Addis Ababa, Ethiopia; 3 International Livestock Research Institute, P. O. Box 30709, Nairobi, Kenya; 4 Present address: Department of Microbiology, Immunology and Veterinary Public Health, College of Veterinary Medicine and Agriculture, Addis Ababa University, P.O.Box 34, Bishoftu, Ethiopia

**Keywords:** Tick infestation, Udder swelling, Evil eye, (mis)-perception, Traditional medicine, Udder health management, Dairy hygiene, Qualitative study, Animal welfare

## Abstract

**Background:**

Ethiopia has high prevalences of udder health problems including clinical and subclinical mastitis across production systems in different livestock species. Previous studies on udder health problems have largely focused on identification of mastitis causing microbial pathogens and associated risk factors. However, relatively little is known about the knowledge and beliefs of livestock keepers regarding udder health problems. An understanding of the beliefs on the other hand would facilitate effective communication between livestock keepers and animal health professionals. Therefore, this study aimed at exploring the knowledge and belief surrounding the causes, clinical signs and treatments for udder health problems in (agro-) pastoral communities in southern Ethiopia using qualitative investigation.

**Results:**

The result showed that udder health problem, locally known as ‘*dhukkuba muchaa*’, which translates to ‘disease of teats’, was classified into three main types: (1) tick infestation (*dirandisa*), (2) swelling of udder often with pus discharge (*nyaqarsa)* and (3) acute mastitis caused by evil eye (*buda*) with ‘bloody milk’. Tick infestation was perceived to directly cause mechanical damage to udder tissue or to resulting in swelling leading to *nyaqarsa*. Our analysis also revealed the strong misperception that acute and severe swelling of udder was caused by evil eye. According to the pastoralists, cows with large udders in the late pregnancy are prone to evil eye infliction upon giving birth. The pastoralists often treat udder health problems by combining both modern and traditional methods. Removal of ticks by hand and acarcide application were the preferred methods for limiting tick infestation while swelling and evil eye cases were treated with antibiotics (e.g. oxytetracycline).

The study also revealed that specific herbs, only known by the herbalists, were used for traditional treatment of udder health. Although this information could not be divulged at the time, it should form the subject of further investigation. Traditional treatment for evil eye was often administered through nostrils, raising questions about its effectiveness.

**Conclusion:**

The narration given by the pastoralists in associating tick infestation with udder health problems was compatible with existing scientific evidences. In this respect, such local knowledge can be better utilized for the educational messages targeting control and management of tick infestation in livestock. However, the misperception of causes for acute udder swelling as evil eye can be problematic as far as the application of appropriate treatment and management of the problem is concerned. The misperception can significantly impact the welfare of animals and highlights the need for capacity building of the pastoralists on the causes and treatment of udder health problems.

## Background

Livestock production is the cornerstone for pastoral and agro-pastoral livelihoods for provision of foods and plays an important economic, social and cultural role [1]. However, significant seasonal fluctuations in feed and water availability, ecosystem changes, extreme weather conditions and presence of animal diseases [2] threaten this important income source. Diseases result in poor livestock health, low productivity, mortality, reduced livestock products and increased risk of disease transmission to humans. When looking at the dairy sector specifically, milk is important in the diet of the pastoralists and can contribute to more than 50% of the energy intake of families, especially in children [1, 3]. In Borana pastoral areas (where the present study was carried out), animals kept primarily for milk production include cattle, camels and goats [4]. In this respect, maintaining the health and productivity of milk producing animals is crucial to ensure good health and nutrition of the livestock keepers.

Udder health problem is one of the major diseases of dairy animals with negative impacts on milk production, milk safety and animal welfare [5]. Mastitis constitutes one of the main udder problems and by definition refers to an inflammation of the mammary glands characterized by changes in the physical and chemical features of milk and pathological changes in the glandular tissue often with non-specific and complex factors responsible for its occurrence. Mastitis occurs in two forms: clinical mastitis with evidence of milk and udder changes upon physical examination and subclinical mastitis only detected by indirect tests such as milk somatic cell count and pathogen isolation [6]. In Ethiopia, a number of epidemiological studies showed high prevalence of mastitis in different livestock species and production systems. For example, animal level mastitis prevalence in dairy cattle was 32.6% in central Ethiopia [7], 64.6% in southeastern Ethiopia [8] and 59.1% in Borana pastoral areas in southern Ethiopia [9]. Studies in the pastoral areas of Ethiopia in camels revealed animal level prevalence of 29.0% [10] and 44.8% [11]. Based on indicator paper test, 15.5% subclinical mastitis prevalence in goats has been reported in Borana pastoral areas [12]. Etiologically, Gram positive cocci are the most common bacterial agents isolated from the milk of animals affected by clinical or subclinical mastitis. Mastitis, especially the subclinical form, causes significant economic losses in dairy cattle. In Ethiopia, the total loss caused by subclinical mastitis associated to *Staphylococcus aureus* was estimated at USD 78.65 per cow per lactation in small-size farms (<5 heads of cattle) and USD 150.35 in large-size farms (*≥*50 heads of cattle) [13].

Past studies mainly focused on identification of microbial causative agents and associated risk factors. However, research focusing on social and cultural aspects of how livestock keepers perceive and manage generally udder health problem (e.g. mastitis) in their livestock is lacking. This information and involvement of livestock keepers is needed in order to implement suitable and effective treatments, control and management of mastitis. Therefore, there is a need to assess knowledge and beliefs of livestock keepers regarding prevailing livestock health problems before designing possible disease control programs through awareness creation and education of livestock keepers. In spite of the importance of milk and milk products in in Borana, detail studies on milk handling and processing are limited. The present study was implemented as part of a research project aiming to improve the handling practices and microbiological safety of milk and traditionally produced dairy products in the area. The study aimed at exploring the knowledge and belief of communities in the area about the causes, clinical signs and treatment of different udder health problems. In pastoral livestock keeping communities women are mostly involved in routine management of milking animals [14] and are supposed to be more familiar with udder health problems and related diseases of dairy animals than men. In line with this, we involved largely women in our study.

## Methods

### Study area

**Fig. 1:**
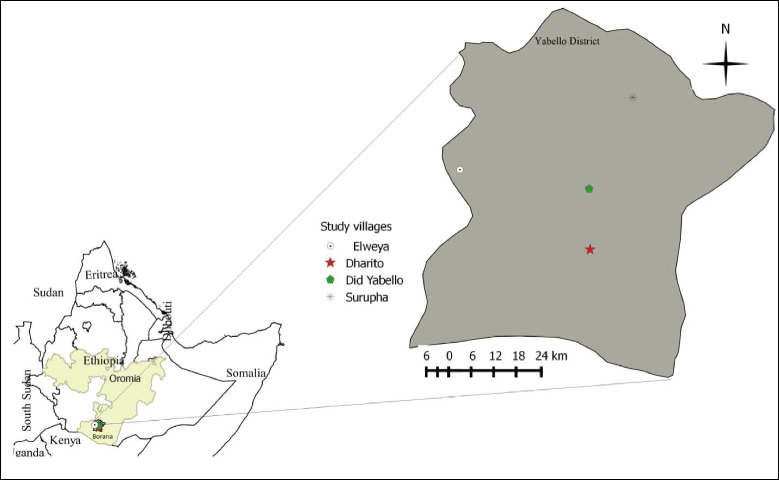
Map of study area (four villages in Yabello District, Borana Zone, Oromia Regional State, Southern Ethiopia) (Note: The boundaries are unofficial)

The present study was carried out in four village administrations (Kebeles) of the Yabello district in the Borana Zone, southern Ethiopia. Borana is located in southern part of Ethiopia bordering Kenya (Fig. 1). The zone has a semi-arid to arid climate with high variability of rainfall resulting in seasonality in the off-take of livestock and livestock products. The rainfall distribution of the area is bimodal with the long rainy season extending from March to May and the short rainy season from September to November. From June to August is the cool dry season while December to February is the warm dry season [15].

The zone has an estimated population of 962,489 (487,024 male and 475,465 female) with 91.2% of the population living in rural areas [16]. The same information source also indicated that 90.9% of the population speaks Afan Oromo as mother tongue and the population follows different religions with 47.3% Protestant Christian, 35.0% traditional, 9.6% Muslim and the remaining other religions. Historically, Borana people are nomadic people residing in southern parts of Ethiopia and northern Kenya, keeping cattle as their main livelihood strategies and seasonally moving in search of feed and water or to escape the risk of potential diseases. Associated with various environmental, social, political and cultural changes, Borana people are diversifying their livelihoods strategies. The livelihood diversification of traditionally cattle keeping Borana families is often evident in terms of growing shift towards crop farming, keeping of other types of livestock such as camels and small ruminants and involvement in non-farming activities such as petty trading, employment as daily labourer and charcoal production [17, 18]. Based on discussion made with people working in Yabello District Pastoral Development Office, animal health delivery systems in Borana pastoral areas include government veterinary clinics/animal health posts and private drug shops. Community-based animal health workers are also active in the pastoral areas of Borana in providing animal health services. The education levels of people involved in animal health delivery vary which include few weeks of training for the community-based animal health workers and formal training for veterinary doctors and animal health assistants. Livestock keepers themselves also buy and administer drugs especially in remote villages. Use of traditional veterinary medicine is also common in the area.

For this present study, four village administrations (Dharito, Elweya, Surupha and Dida Yabello) were selected based on ongoing research projects aiming to improve the small ruminant value chain and the high milk production potential of the villages. The qualitative data collection was carried out in July 2015 with subsequent substantiation of unclear concepts through informal discussions with the pastoralists in December 2015.

### Methodological approaches

Different participatory data collection methodologies which included individual semi-structured in-depth interviews (IDI), focus group discussions (FGD), informal discussions with key informants and observations were used in the present study. Individual interviews were held with 40 women (10 in each village) using a semi-structured questionnaire which was initially developed in English and then translated to Afan Oromo (Borana dialect), a language widely spoken in the study area. As background information, age of the respondents and types of livestock they were keeping was first asked in the IDIs. For the main study, the questions mainly focused on the knowledge and perceptions of pastoralists about causes, clinical signs and treatments of udder health problems in different livestock species. The questions guide was pretested for clarity by interviewing three women before the actual study started.

In Borana, several households, mostly belonging to same lineage, reside in clusters or neighbourhoods (called *olla*). The respondents for individual interviews were identified by driving through the villages and stopping in different *ollas*. The selection of the respondents was made with assistance of development agents in each village facilitated by a female field assistant from Yabello District Pastoral Development Office with a background in animal production and more than 4 years of working experience in dairy production in the area. The respondents were married women (having major domestic responsibilities) and who own lactating animals at the time of the fieldwork or during the previous rainy season. The interviews were held either under the shade near to the houses of the pastoralists or inside their houses. When the women were first approached for interviewing, most of them were hesitant and even not happy to exchange greetings. However, upon explaining the objectives of the study, all agreed to be interviewed and consequently none of those approached declined the request for participation. All interviews were carried out by the female field assistant in the presence of the first author except in few cases in which men were around and informal discussion with them was necessitated to divert their attention and to minimize distraction during the interviews with women. The interviews were documented using audio recording and field notes.

Following the IDIs, FGD with women in each village on similar topics was held to verifying already obtained information and to obtain further information about udder health problems. To select participants for the FGD, the development agents identified 6–8 women, preferably, from different *ollas* and representing different age groups. The FGD was then held at a central place agreed upon in advance. The group discussion was moderated by the first author with help of the female field assistant. After the interviews and FGDs, informal discussions with the participants or people encountered along the way were held on general aspects of milk handling practices and udder health problems to triangulate and complete information obtained.

Before the interviews and FGDs, verbal consent was obtained from each of the respondents after explaining the objectives of the study and assuring anonymous use of the information. Verbal consent was considered enough given that the information collected in the present study was assumed to be what is shared commonly among the pastoral communities and no personally sensitive information was collected. As part of good practices in undertaking the research project, the respondents were given chances to ask the interviewer on any of the topics discussed. After that brief feedback on the general milk hygiene and causes of mastitis were given to the respondents.

### Data management and analysis

The audio recordings of the interviews and discussions were transcribed and translated into English. The data was analysed qualitatively by repeatedly reading the transcript to identify different themes, which were coded using the free software QDA Miner Lite v1.4.3 Provalis Research [19]. Essentially, the themes were identified from the questions guide and further emerging themes were identified iteratively starting from data collection throughout the analysis process. Content analysis was carried out for clinical signs of udder health problem described by the pastoralists to identify commonly used terminologies used by the pastoralists in describing the problem. To portray the qualitative data different quotes in the words of the respondents were highlighted.

## Results

### Background information (age of respondents and livestock production practices)

The reported age of respondents involved in the individual interviews varied from 17 to 50 years (average of 32 years, median = 30 years). Cattle, camels and goats were the common livestock species kept for the purpose of milk production in Borana. Out of 40 women interviewed individually 39, 33, 13 and 4 women reported keeping of cattle, goats, camels and sheep respectively. Similarly, the milk from the different livestock was not equally preferred by the people. The widely produced and consumed milk in the area was cow milk. Borana pastoralists highly value cattle both for economic and cultural reasons. The following quotes in the words of the pastoralists strengthen above statements.

*“Cattle are the foundation of the people here; milk is also mainly obtained from cattle” (IDI 7).*

*“Borana started rearing camels very recently. What we know more is about cattle” (FGD 3).*

Keeping of camels for milk production was preferred by the pastoralists due to the large volume of milk camels produce especially during dry season.

*“Good milk is from cow (tasty) but large volume [is] from camels” (IDI 8).*

Goat milk was consumed especially by mixing with tea and it was perceived to have better nutritional value as indicated below.

*“Though goat milk is small in volume, when we want to dilute with water, goat milk has more vitamins* [to indicate nutritional value*]” (IDI 3).*

### Themes identified under udder health problems and treatments

**Fig. 2:**
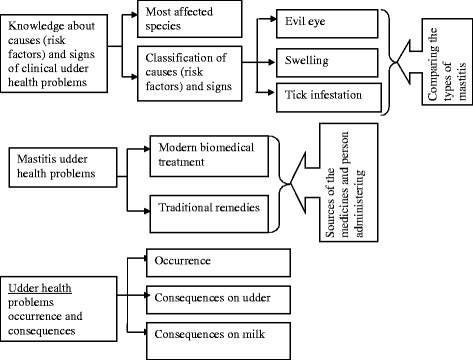
Hierarchy of major themes discussed during the interviews and group discussions

The major themes identified in the present study are indicated in Fig. 2. The concepts of causes, risk factors and clinical signs were intermingled and as a result the identified themes were grouped together in the three types of udder health problems reported.

### Knowledge about causes and signs of clinical udder health problems

Locally, udder health problem is known as *dhukkuba muchaa*, which literally means ‘disease of teats’. Though the name implies “disease of teat”, the term is understood to be a general udder health problems. Pastoralists associated the problems of udder health with different factors and grouped based on the perceived causes and clinical signs into different categories. The main categories identified were: *dirandisa* (tick infestation), *nyaqarsa* (chronic swelling in the form of a boil) and *buda* (which means evil eye and is characterized by bloody milk). The qualitative investigation revealed that there was no clear differentiation between the different types of udder health problems and the multifactorial causes was well appreciated by the pastoralists as is illustrated by the following quote:

*“Udder health problem results in swelling of udder, blood can come with milk, ticks can infest the udder and the teat; it can cause swelling. Udder health problem is caused by different factors” (IDI 8).*

In some cases differences in the predisposition of the different livestock species was noted. For example, it was stated that udder health problems are common in cows compared with goats. It was described that udder health problem is severe in camels compared with cows.

*“Udder health problem occurs in both cattle and goats, udder becomes ill when trauma due to thorny plant (goraa), painful when calf suckle and to prevent the painful situation, smeared with butter. Udder health problem is common in cows compared with other species” (IDI 24).*

*“Camels are highly affected by udder health problem. It is camel which is highly affected by buda or swelling. When camels are affected by mastitis, no survival of the calf. It is not like cows. All [teats] affected once. Regarding mastitis, camels are highly harmed by mastitis” (FGD 2).*

Content analysis of the interviews and the discussions about clinical signs indicated that the most common term used to characterize udder health problem was ‘swelling of udder’ followed by ‘bloody milk’ (Table 1).

**Table 1:** Clinical description of udder health problems by pastoral people: results of content analysis of individual interviews and focus group discussions

Clinical description of mastitis	Number of times mentioned^a^
Swelling of udder	33
Bloody (red) milk	16
Pus discharge upon milking	9
Blind teat	8
Painful udder and teat	6
Difficulty in milk letdown	3
Teat perforation	3
Milk with stingy odour	2
Cracks on teat	2
Abnormal discharge from teats	1
Skin sloughing of teats/udder	1

^a^Number of the issue was mentioned (can be multiple times in the IDI, FGD or informal discussion)

### Tick infestation (diraandisa)

The pastoralist women mentioned the predisposition of domestic animals for udder health problems due to tick infestation and they explained well the biology of ticks during the interviews. They mentioned that tick infestation causes swelling of udder, wounds on the skin of udder at the attachment site and leads to blind teats.

*“…tick, small creature, moves on the ground and can infest cattle. Causes wounds on the animal” (Informal 2).*

*“The main cause is tick infestation. Whether cattle, goats or camels, tick infestation is the one which causes major problems” (Informal 1).*

*“Tick infestation can cause udder health problem. Even if you remove [the tick], the mouth can remain [in attachment site] and lead to ill health. Also cows can acquire and experience chronic swelling of udder” (IDI 14).*

*“There is also tick infestation which causes swelling and blockage of teat” (IDI 40)*

### Swelling (nyaqarsa)

Udder health problem characterized by swelling of udder was referred to as *nyaqarsa* which literally translated means a boil. As per the description by the pastoralists, *nyaqarsa* does not involve diffuse inflammation of the udder potentially as a result of ascending bacterial infection through teat canal. It was often recognized as a distinct disease of udder but the cause of *nyaqarsa* was associated with different factors including tick infestation and trauma by thorny plants. The respondents both in IDI and FGD described that swelling (nyaqarsa) bursts leading to wound and teat loss.

*“Nyaqarsa is changed to ilke [swelling with pointed tip]. It becomes hard. If blood and pus is not coming out (discharged),, the teat opening becomes hard and blocked” (FGD 1).*

*“Nyaqarsa, is like a boil, causes swelling. Whether medicine is applied or not, nyaqarsa can burst. Wound is formed and when washed with medicine can be healed. It is also this one which causes discharge from teat” (FGD 4).*

*“Udder becomes ill because of trauma due to thorny plant” (IDI 24)*

### Evil eye affliction *(buda)*

Pastoralists perceived evil eye affliction of the udder as one of the main causes of udder health problem. The belief that there are people whose eyes can change ‘white milk’ into ‘red milk’ when they stare at the udder of animals, especially on animals with good milk production, is very common. According to the respondents cows with large udder during late pregnancy are highly vulnerable to evil eye infliction after giving birth during early lactation. Most interviewees expressed the idea that acute udder health problem (more referring to acute mastitis) is caused by evil eye and they believe that it is caused by looking intently at animals. In the words of one informant:

*“When cows are pregnant and ready to give birth, there is something in the udder which gives high milk yield. There is ‘evil eye’ which sees the milk in udder and kajeellaa [envy], causes swelling of udder. There is milking of blood. There is milking of blood. You see blood like when slaughtering [animals]. There is discharge of blood from cow teats” (Informal 2).*

*“Evil eye afflicts when the udder of pregnant cows increases in size” (IDI 38)*

According to the pastoralists, people with evil eye have super ability to see milk inside the udder of animals. As per the descriptions of the pastoralists, “seeing the milk by the people possessing evil eye” was not the observation obvious to anybody (e.g. differentiating lactating and dry cow) rather it was more referring as if one observes milk in a bucket after milking including the colour.

*“There are people which are by nature buda. It is said that there is a person who can see milk inside udder” (IDI 28)*

In addition to causing udder health problem in livestock, evil eye was widely believed to cause many health problems in animals or humans and crop failure. It is believed that some people naturally possess evil eye but such information is shared secretly among the community. According to the respondents, saying to somebody “you have *buda*” is an insult and can lead to a disagreement to the extent that who said so can be legally held accountable for defaming the person.

Another related cause of udder health problem was envy (*kajeellaa*), literally referring to an intense desire or need for other people’s livestock, crop or property. *Kajeellaa* was believed to aggravate the affliction by evil eye. For example, when someone’s belongings with superior quality (could be livestock or crop) are widely praised among the community, this may lead to evil eye among the people getting the information (hearing) and cause the damage. According to the description given in group discussion, the one who is praising about the animals can be innocent (without evil eye) but a person possessing evil eye may hear the information and subsequently inflicting the damage. If information about good milk production of animals is shared and reached 3 or 4 people, all of the people may not be free of evil eye ability. The problem of *kajeellaa* on milk quality was described by a participant in one FGD as follows:

*“Three of our cows became pregnant and people started talking about their pregnancy. The whole neighbourhood started talking about by saying: Oh! ‘Your cows are pregnant and how big their udders are!’ The cows gave birth and after that their milk stayed for 9 days without forming curd [i.e. without solidification). Thereafter, I requested the neighbours ‘to spit for me’ (tufaa). By putting the medicine for buda [referring to spitting] in the milk, it started to curd” (*FGD 1*).*

The pastoralists also believe that evil eye is responsible for reduced milk production of animals and calf rejection by cows after birth (*finqilchaa*).

### Treatment approaches of udder health problems

Various treatment approaches were used for the treatment of the different types of udder health problems, including modern and traditional treatment approaches depending on the types of the problems. According to most of the interviews, people tend to use modern medicine for *nyaqarsa* and traditional medicine for evil eye affliction. As revealed during the FGDs, there was no consistency in the order of the use of modern or traditional medicine for treating udder health problem. The overall idea during the FGDs showed that people tend to resort to either traditional or modern medicine when the animal being treated is not responding to the first treatment regime they are using. This was clearly evident from the following quotes:

*“When Borana [traditional] medicine is not effective (does not cure), we try black medicine (taramishi) [oxytetracyline]” (FGD 2)*

*“We try with black medicine, if not successful revert to Borana medicine” (FGD 3)*

*“When Borana medicine is found not effective (unable to cure), the thing [milk] is not coming out and the milk becomes watery like whey; we injected black medicine* [oxytetracyline]*; also the white one* [penicillin and streptomycin]*. We use combinations of the medicines” (FGD 2).*

### Traditional treatment

The traditional treatment termed as *qorsa* Borana (means Borana medicine) varied from the use of herbs to procedures involving magical practices. Specific plants, only known to the herbalists and not revealed to the users, were used for the treatment of udder health problems. Especially for acute udder health problem caused by evil eye with discharge of blood mixed in milk, traditional treatments were used. The term *walda* was often mentioned by the pastoralists referring to a specific plant used in the traditional treatment of udder health problem and other livestock diseases. However, it was evident from the interviews and discussions that other types of plants were also used.

*“Walda is used. We don't know the plant name and we get from healer” (IDI 5).*

The pastoralists described that different modes of application of the plants materials were practiced which include dropping the juice of the plant after chewing into nostril or mouth, smearing of the plant on udder or teat after burning and mixing with butter, fumigation of udder with the smoke of burning plant, rubbing with the dying burning wood (*barbadaa*). The intention of applying herbal medicine was either curative or preventive. It was mentioned that traditional medicine can be applied before animals giving birth to prevent occurrence of udder health problem or after delivery to treat clinical cases.

*“After chewing the medicine, the juice is dropped into the nostril. After chewing it is also spitted on the udder. The udder of a pregnant animal is also smeared. It is also smeared after burning of the plant. There is a difference when applied before birth and after birth” (FGD 2).*

*“There is medicine which is dropped into the nostrils of lactating or late pregnant cows. There is also medicine smeared on the udder before delivery. To prevent blockage of teats at delivery, traditional medicine is smeared on [udder] in animals having large udders during late pregnancy; also after birth” (IDI 36).*

The common magical practice employed to cure animals attacked with evil eye was called *tufaa* (literally means spitting). *Tufaa* was used for different health problems (e.g. mastitis, calf rejection or low milk yield or failure of milk to curdle) if evil eye was thought to be the cause. Spitting as countermeasure against evil eye affliction is done by requesting the person suspected of possessing evil spirit and who might have caused the animal health problem to spit on the animal. If the suspected person agrees, he/she will spit on the animal by reciting the phrase: ‘if I have eaten (inflicted) you [referring to the animal], may God recover you from the problem”. According to the interviews and discussions, the practice of spitting for the treatment of udder health problem was found to be declining compared to past times due to fear of people to ask the suspected person for spitting. Nowadays, referring somebody to possess evil spirit (*buda*) is becoming an offence for people and in some cases can lead to disagreement. Due to this, alternative means of the procedure of spitting was practiced without pinpointing to a specific person. The person whose animal has been diseased calls all people in the neighbourhood to spit and all people state publicly that they have not attacked the animal. In the words of a discussant and an informant in the informal discussion, respectively:

*“That is in the very past. Currently this practice does not exist. If you now say to somebody, “you are buda and it is you who ate my animal”, you may face serious legal consequences. You can be imprisoned. Even if when the heifer is affected by buda and you know very well and [the heifer] entered to the house of the person [indicating that the animal was inflicted by the person], you cannot claim anything. You cannot say to the person you ate my heifer and please come and spit for me. In that case you can be imprisoned and no way to come out of prison. No way to ask. You simply leave when the cow is diseased, teats blocked or calf died [due to buda]. [Nowadays], you cannot say that ‘you have buda’* [to an individual]*. In that case, the person can kill you” (FGD 1).*

*“In the past (long time) Borana would call the person who was known to be buda and requested from him/her by saying: ‘it is you who ate my livestock and now spit for me [laugh…]. It is you who ate and now come and spit for me’. The person is obliged to spit. Nowadays, that is not the case. You are scared to ask the person. That can have legal consequences. It is not said anymore, spit for me. When you suspect somebody among community members, you call all people and request to spit. You say, we don't know who ate, so all come and spit. There can be the suspect among the community. The people spit by saying: ‘if it is me who ate you, get well’. Thereafter, the milk changes from blood to milk. Nowadays, this practice [requesting a person for spitting] is not common. If you say that the person can go to court and can lead you to serious legal consequence. As a result, the owner of the affected animal lost his/her animal and simply decline to complain” (Informal 2).*

An alternative practice to spitting to counteract the effect of evil eye and simultaneously minimize the possible disagreement was to give milk to the suspected person without his/her knowledge. In an interview it was stated that:

*“When you suspect somebody, you don't complain and not let the person know you are suspecting [*him/her*]. You simply invite [*him/her*] for milk and you just pour milk into a cup and give to the person to drink. After that it is considered that the person has spitted for you” (IDI 15).*

Traditionally *nyaqarsaa* was treated by incising the swelling and removing the pus. Instead of using traditional medicine, nowadays people tend to resort to modern medicine for the treatment of *nyaqarsa*.

Traditionally, tick infestation was treated by manual removal. The qualitative analysis showed that the different treatment and control strategies of tick infestation are well known among pastoralists and that there was agreement that tick associated problems have been reduced compared to earlier times. A typical example illustrating this is the exact quote below from informal discussion with a pastoralist in the area:

*“Now ticks are not affecting udder. People are wise and use acarcides (skin medicine). This medicine eliminates ticks. There had been changes with regard to this. When you inject medicine, ticks fall down” (Informal 1)*

### Biomedical treatments

Borana pastoralists use different modern drugs for the treatment of udder health problems as mentioned during the FGDs. The two common drugs mentioned by the pastoralists were ‘black’ and ‘white’ medicines, literally referring to oxytetracycline and penicillin-streptomycin (PenStrep), respectively. Normally, the pastoralists use the medicines not only for udder health problems but also for other livestock diseases. It means that intramuscular was the commonest route of administration. The animals were either taken to a veterinary clinic or the pastoralists buy drugs and treat by themselves. In the words of the respondents:

*“We buy from market and have at home. Black medicine is for buda. Black medicine is for many [health] ailments. When udder swells, injection is given on two sides [of rump). It regresses the swelling” (FGD 3).*

*“The udder swells. When udder swells, black or white medicine is injected by ourselves or the animal taken to clinic …” (FGD 4)*

*“Before causing swelling it can be removed using medicine for scabies [*acarcides*]. With the injection of the medicine, ticks disappear” (FGD 3).*

As the following quotes of the group discussions in different villages show, the participants indicated that people have reverted to modern medicine from traditional medicine for treatment of udder health problem:

*“Mostly people use black medicine. It is not like earlier times. Except [newborn] rejection [by dam], swelling of udder is treated with black medicine” (FGD 2).*

*“Nowadays we are using modern medicine. We use black medicine. When udder swells, we inject with medicine” (FGD 1).*

*“Nowadays after healthcare came, we take [livestock] to clinic like human and let them be treated using various drugs and injections” (FGD 4).*

The acaricide mentioned by the pastoralists and used for treatment of tick infestation was termed as *cululuqaa* (shining medicine) which apparently refers to subcutaneous injectable ivermectin.

### Occurrences and consequences of udder health problems

Most informants indicated that mastitis leads to various problems of the udder and teats, weak newborns and milk quality or safety problems. The most frequent recurring theme regarding the consequences of udder health problem was loss or blockage of teats. According to the informants, loss of teats leads to early elimination of animals by selling.

*“When teats blocked, the animals is sold and changed” (IDI 17).*

The other consequence of udder health problem stated was discarding of the milk of the affected animals due to aesthetic reason. Especially, the ‘bloody milk’ of acutely affected animal was not consumed by the pastoralists and either left not milked, milked on the ground or the milk given to dogs.

*“It is not milked, unless milked on the ground. It has problem for calf and humans. Milked on the ground. Nobody drinks until becomes normal. Milked and given to dogs. Dogs drink” (FGD 1)*

However, in the FGDs there was no consensus among the pastoralists regarding the use or discarding of the milk from animals with udder problems. It was revealed that some pastoralists use the milk after boiling or by adding into boiling tea. The pastoralists also explained the effect of udder health problem on milk quality and difficulty of processing milk into different products. The pastoralists traditionally process milk into different products such as yoghurt (*ititu*), butter, ghee and butter milk. *Ititu* is traditional milk product widely consumed in the area and prepared by natural fermentation through accumulating whole milk for several days to weeks and regularly removing the whey (the fluid part). The effects of udder health problems on milk quality and processing were explained by the pastoral women as follows.

*“The milk smells extremely stingy even after returning to white. Not good for animals as well as humans. Irrespective of this, it is produced by people and you cannot discard. This thing is consumed. There is nothing, Borana cannot consume” (FGD 2)*

*“[Milk from animal with udder health problem is] not for drinking. It is boiled in a pot and added to tea. It is not consumed fresh. This milk is not even suitable for churning. Even if you churn it does not yield butter. If you also make ititu (yoghurt), you cannot drink. It has stingy odour. You boil like water and add to tea. We don't discard. We prepare (boil) like that and drink” (FGD 2).*

*“Now there is a problem with the milk. When milked, the milk is not used fresh. It clots/precipitates. It can be yellowish if it stays for some time. It cannot form [proper] curd easily, if you want to change to ititu (yoghurt)” (*FGD 2*).*

The other consequence of udder health problem perceived by the pastoralists was the negative effect on health of the newborn animals as a result of consuming the milk of the affected dams.

*“The calf can be affected by disease characterized by hair falling when suckling the dam affected with evil eye. … No appetite for grazing and then death. When the calf suckles, diarrhea, extreme loss of body condition and death” (FGD 1)*

## Discussion

The present qualitative study of the knowledge and beliefs of pastoralists about the cause, clinical signs and treatment of udder health problem is part of a research project aiming to improve the handling practices and microbiological safety of milk and traditionally produced dairy products in Borana pastoral area in Ethiopia. Employing of different participatory qualitative tools in the present study enabled us to describe the details of the knowledge of pastoralists about udder health problem and current treatment practices in different livestock species. Thanks to qualitative research methods much more detailed information on attitudes and beliefs could be obtained compared to quantitative approaches.

In Borana, the term “dhukkuba muchaa” used by the pastoralists to refer to udder health problem in general and specifically mastitis was not exactly matching the western disease nomenclature. In Oromo language, ‘dhukkuba’ means disease or illness and ‘muchaa’ means teat (so literally means ‘disease of teats’). The name for udder in Afan Oromo is ‘gurruu’. Similar studies also indicated such differences in disease naming between African traditional medical practices and western sciences. On the other hand, udder health problem specifically mastitis was understood by Borana pastoralists to be caused by various factors; often the factors acting collectively in causing udder health problems. For example, the pastoralists had understanding that injuries by thorny plants cause udder problems. Such understanding is compatible with western scientific explanation of the multifactorial causation of mastitis [20]. On the other hand, it should be noted that the description given for mastitis by the pastoralists can be potentially periparturient edema and physiological discoloration of milk as result of colostrums which should be clarified through creating close collaboration with the pastoralists.

Quality and effectiveness of animal health management in small-scale livestock production system is affected by farmer and farm characteristics, and economic, institutional and biophysical factors. The education level and experiences of livestock keepers largely influences animal health management [21]. In this respect, pastoralists have good knowledge about the health of their animals with a huge potential for integrating their knowledge with the veterinary service delivery system to solve prevailing livestock health problems [22–24]. This is due to the fact pastoral people usually live in close proximity to their livestock and do have lifelong experiences about livestock production and diseases management. Therefore it is no surprise that sometimes pastoralists can have animal health knowledge and skills comparable to trained animal health professionals. For example, in a study by Catley [23] there was a good agreement between pastoralists’ and veterinarians’ disease names and diagnostic criteria.

On the other hand, even if detailed studies are lacking for animal health, studies in human health showed that there can be strong cultural beliefs about diseases which can subsequently be a barrier for seeking treatment and provision of effective health services [25–28]. For example, Masika et al. [29] in their studies about the local tick control strategies by cattle farmers in South Africa indicated that very few farms associated ticks with tickborne disease such redwater and Gall sickness (anaplasmosis). The authors [29] further noted that wrong understanding of the causes and transmission of diseases can lead to ill-directed treatments and widespread deviation from the recommended directions of use when administering conventional medicines. It was recommended that this should be addressed by farmer training and supplying appropriate information [29]. Similarly, the present study revealed that the pastoral women blame acute udder health problem on evil eye since there is a lack of knowledge on causative agents and how the causes (pathogens) are transmitted.

Scientifically, a number of microbial agents are responsible in causing mastitis in livestock with presence of multitude of predisposing factors which include: animal risk factors (age, udder/teat conformation), environmental risk factors (poor housing condition) and pathogen risk factors [6]. The causes of udder health problems in Ethiopia may be very different from Western developed countries. In the case of Ethiopia in which livestock are largely kept under extensive production system, local damage and infection from superficial skin wounds seems to be a large contributing factor [10]. In the case of Western countries (largely intensive management system), ascending bacterial infection through the teat canal can be a driving factor for udder infection [6]. This would have implications for prevention and treatment strategies under Ethiopian production systems in which greater emphasis should be put on skin inspection and wound care. Prevalence of contagious pathogens such as *Staphylococcus aureus* and *Streptoccus agalactiae* have been significantly reduced in importance in developed countries [30] while still a big challenge in the production systems of developing countries like Ethiopia [8–10, 12].

In Ethiopia, linking evil eye and health problems is a widespread belief [31, 32]. Our findings that pastoralists link evil eye with acute udder health problem characterized by inflammation of udder is consistent with the findings of a study conducted by Mesfin and Shiferaw [33] on the indigenous veterinary practices of South Omo (agro)-pastoralists of southern Ethiopia. Similar to Borana people, the different ethnic groups in South Omo link evil eye with mastitis and apply various traditional treatment approaches such as spitting and smearing of traditional medicine on the udder. A study by Teferra and Shibire [34] in Borana also showed linking of severe human mental illness with heterogeneous supernatural causes such as evil spirit, curse and bewitchment. In a study in southwestern Ethiopia among people suffering from tuberculosis, it was found that 50% of tuberculosis suspects (with signs of cough for at least 2 weeks) linked the cause of tuberculosis with evil eye [31]. Similarly, in other African countries the belief in evil eye as cause of various health ailments is also a common situation. In this respect, Comoro et al. [35] reported strong beliefs of mothers in linking severe malaria among children with supernatural causes such as evil spirit and bewitchment, and mild malaria with biological cause (mosquito bites).

In our present study, the misperception of acute inflammation of udder as evil eye can be problematic in the management of mastitis and lead to poor welfare of the animals. Traditional treatment for evil eye is often administered through nostrils and this procedure may not be effective against the disease. Moreover, resorting of the pastoralists to traditional magical treatment (e.g. spitting) can have negative impacts on welfare of the animals and cause further economic losses. The delay in appropriate treatment of mastitis can lead to loss of teats due to progression of mastitis to terminal stage and leading to early elimination of animals [6, 20]. The practice of spitting for the treatment of udder health problem was found to be declining compared to past times due to fear of people to ask the suspected person for spitting. This can be a good strategy to teach the pastoralists to follow proper management of udder health problems.

Though traditional medicine can complement modern medicine, antibiotic treatment remains necessary to achieve bacteriological cure of infection [35]. Cure rate of mastitis treatment depends on a number factors related to host, pathogens and treatment regimen. For example, it is not recommended to treat mastitis caused by *Staphylococcus aureus* infection in older animals, chronic infections, or penicillin-resistant isolates [36] and such possibilities should be also considered when the udder health management programs are going to be designed in the pastoral area. There is now a move in developed countries to leave some forms of mastitis untreated (e.g. those caused by *Escherichia coli*) due to the pressure to reduce antimicrobial use in livestock production. This may not be applicable in Ethiopia, where most mastitis is associated with gram-positive species [8–10, 12].

Important to note, though, is the apparent lack of topical intra-mammary antibiotics in these regions and instead injections are used which may not be optimal as a treatment option. In the present study we could not find evidences indicating availability of drugs specifically intended of the treatment of udder health problems (e.g. intrammammary infusion form). The pastoralists were dependent on intramuscular injectable antibiotics for the treatment of general animal health problems and the commonly stated drugs were intramuscular injections of oxytetracycline and penicillin (often combined formulation with streptomycin). Some studies showed better efficacy and increased bioavailability in udder when intramammary infusion is used compared with injectables; which further can minimize the potential occurrence of antimicrobial resistance [37]. Moreover, previous studies elsewhere showed that oxytetracycline is ineffective for mastitis treatment [38–40]. In this regard, further strategies should be designed by local government to avail efficacious antimicrobials in correct formulation for the treatment of mastitis in the area. But considering the low hygiene standards around milking and udder health in the Borana community, well planned training should be given to the animal health professionals as well as the pastoralists before implementing such treatment regime. In intramammary treatment, strict hygiene must be followed by careful cleaning and sanitation of the teats before infusing to avoid the introduction of pathogens [6]. During intrammary infusion the delicate tissues lining the teat duct can be traumatized with further complications and due to this incorrect procedure is one of the factors responsible for mastitis treatment failures [41].

Therefore, improvement of milking hygiene is the aspect which necessitates due consideration is preventive measures for mastitis. Poor milking hygiene in terms of not washing and drying udder, and milkers’ hand before milking had significantly increased the prevalence of mastitis in dairy cattle [8]. The high prevalence of mastitis causing contagious pathogens (e.g. *Staphylococcus aureus*) likely is a direct result of poor hygienic practices. Previous studies in Ethiopia showed that among the different bacterial pathogens isolated from cases of mastitis, *Staphylococcus aureus* (a contagious pathogen) represents the commonest one. For example, 24.1% [8] and 41.5% [7] of the bacteria isolated from cows suffering from mastitis were *Staphylococcus aureus*. Similarly, *Staphylococcus aureus* has been isolated from 26.3% [10] and 12.8% [12] of camels in pastoral areas of Ethiopia; in both reports showing the highest proportion among the different pathogens. During our present fieldwork, it was observed that most pastoralists do not follow hygienic precautions such as milking of mastitis affected animals at the end and washing hands between milking different animals, thus enabling cross-contamination from diseased to healthy animals. Since pastoralists are not aware of pathogens and how they are transmitted during milking, the role of hygiene during milking is not obvious. Therefore, awareness creation on good hygienic and milking practices is necessary to reduce the high incidence and impacts of udder health problems in dairy animals in the area.

Interestingly, the pastoralists associated udder health problem with tick infestation which is compatible with existing scientific evidences. They correctly described biology of ticks and the damage ticks cause on udder or teat with subsequent occurrence of mastitis. Ticks can cause direct physical damage on the skin of the udder or teat favoring bacterial infection and occurrence of mastitis. Different epidemiological studies identified high prevalence of subclinical mastitis associated with the presence of lesions on the skin of udder or teat of cattle [7, 8] and camels [12] in Ethiopia. Abera et al. [10] also reported higher prevalence of mastitis in animals having udder or teat lesion and infested with ticks in camels in eastern Ethiopia. Thus, optimal control of tick infestation through acaricides application or careful hand removal in dairy animals can reduce the prevalence of mastitis and the resulting economic losses.

The negative consequences of mastitis on milk quality, animal productive longevity and the difficulty in milk processing were well described by the pastoralists, and in line with the results of other scientific studies [20, 42]. Mastitis directly affects milk composition often reducing lactose, non-fat solids and total solids [43] which can compromise milk processing and causes low yield of milk products. The change in milk composition can have direct negative consequences for milk quality and nutritional security of the people.

Udder health problem is an important disorder of dairy animals reducing milk production and subsequently causing food safety and food security problems in livestock keeping communities. The effect of mastitis on food and nutritional security of pastoral community is more serious due to the facts that milk and milk products largely contribute to the diet of the communities and is essential for child nutrition.

## Conclusions

The good understanding of the pastoralists about the association of tick infestation with udder health problems can be better utilized in the preparation and implementation of educational messages targeting skin problems and udder health. On the other hand, the misperception of evil eye as a cause for acute udder inflammation and administration of traditional treatments through nostrils should be focus of future attempts to improve the awareness level of the pastoralists. Therefore, there is clearly a need to design effective and culturally sensitive communication and capacity development strategies to increase knowledge on causes of udder health problems, on proper treatment, management and prevention, and in general good milk production practices. Our study also revealed that specific herbs, only known by the herbalists, were used for traditional treatment of mastitis and this aspect should also be further investigated to determine their effectiveness.

## Abbreviations

FGD: Focus Group Discussion
IDI: In-depth Interviews
Informal: Informal discussion
